# Empirical Research on Acceptance of Digital Technologies in Medicine Among Patients and Healthy Users: Questionnaire Study

**DOI:** 10.2196/13472

**Published:** 2019-11-29

**Authors:** Sabur Safi, Gerhard Danzer, Kurt JG Schmailzl

**Affiliations:** 1 Center for Connected Health Care UG Medical School Brandenburg digiLog Neuruppin Germany

**Keywords:** innovative health care applications, e-Health, Technology Acceptance Model, health care innovation, electronic medical records, ePatient Survey, sex differences, medical technology

## Abstract

**Background:**

In recent years, interest in digital technologies such as electronic health, mobile health, telemedicine, big data, and health apps has been increasing in the health care sector. Acceptance and sustainability of these technologies play a considerable role for innovative health care apps.

**Objective:**

This study aimed to identify the spread of and experience with new digital technologies in the medical sector in Germany.

**Methods:**

We analyzed the acceptance of new health care technologies by applying the Technology Acceptance Model to data obtained in the German ePatient Survey 2018. This survey used standardized questionnaires to gain insight into the prevalence, impact, and development of digital health applications in a study sample of 9621 patients with acute and chronic conditions and healthy users. We extracted sociodemographic data and details on the different health app types used in Germany and conducted an evaluation based on the Technology Acceptance Model.

**Results:**

The average age of the respondents was 59.7 years, with a standard deviation of 16 years. Digital health care apps were generally accepted, but differences were observed among age groups and genders of the respondents. Men were more likely to accept digital technologies, while women preferred coaching and consultation apps. Analysis of the user typology revealed that most users were patients (n=4041, 42%), followed by patients with acute conditions (n=3175, 33%), and healthy users (n=2405, 25%). The majority (n=6542, 68%) discovered coaching or medication apps themselves on the internet, while more than half of the users faced initial difficulties operating such apps. The time of use of the same app or program ranged from a few days (n=1607, 37%) and several months (n=1694, 39%) to ≥1 year (n=1042, 24%). Most respondents (n=6927, 72%) stated that they would like to receive customized health care apps from their physician.

**Conclusions:**

The acceptance of digital technologies in the German health care sector varies depending on age and gender. The broad acceptance of medical digital apps could potentially improve individualized health care solutions and warrants governance.

## Introduction

Globalization has mitigated a technological change in the health care sector. Thus, the application of digital technologies in medicine is becoming increasingly important. Internet-based health care applications include electronic health records, electronic prescriptions, and digital organization structures in the health care sector [1]. The introduction of digital technologies in medicine faces specific barriers such as end user acceptance [2]. Acceptance is understood to be the result of perception, a concluding evaluation, and a final decision, which leads to a specific attitude or voluntary action. The Technology Acceptance Model (TAM), developed in the late 1980s to study the use of digital technologies by employees, is a standard model to conduct acceptance research in the medical sector [3].

According to TAM, the user’s intention to employ a new information system is influenced by his or her perception of its benefits and accessibility [2,4,5]. In other words, it identifies two main factors that determine the acceptance of an app: “perceived usefulness” and “perceived ease of use” [2,4]. The TAM is derived from the Theory of Reasoned Action, which aims to explain the behavior of users. It may serve to derive predictions about the end user acceptance and to evaluate and confirm already accepted apps [6,7]. The TAM has been developed continuously in the course of further research [2,8,9]. Because the previous theoretical models were incomplete, Lee and Coughlin (2015) developed a 10-factor model as an integrative approach to represent a total of 10 factors that may influence acceptance, namely value, usability, affordability, accessibility, technical support, social support, emotion, independence, experience, and confidence [10].

The acceptance of new technologies in medicine may facilitate access to health-related information or health care services and communication and may thereby significantly reduce errors and costs [2]. Identifying the crucial factors that constitute barriers may be decisive to ensure the acceptance of such innovative technologies [11]. It may also increase patient safety and ensure patient-centered care [12]. For example, an app such as an online patient record is directly linked to the improvement of Germany’s health care system in terms of its integrated care, general practitioner–centered care, and outpatient specialist medical care. Thus, the goal of the implementation of such a record is its integration into the health care system, enabling appropriate and timely decision making and treatment [12].

The purpose of this study was to employ TAM to evaluate datasets pertaining to the use of digital technologies in medicine in order to determine their acceptance in Germany. The study was based on the ePatient Survey 2018, a market research assessment tool employed to evaluate the target groups of medical digital technologies. This survey entails questions on digital skills, user profiles, and possible apps and aims to generate a representative picture of the acceptance and spread of new digital technologies in medicine to derive respective practical recommendations for action [13].

## Methods

### Research Philosophy, Design, and Strategy

The ePatient Survey is one of the most comprehensive online surveys in the German-speaking region and has been conducted annually in the digital health sector since 2010 [13].

The survey uses standardized questionnaires to provide information about the prevalence, impact, and development of digital health apps. These questionnaires are distributed to patients across Germany through health-related websites, newsletters, and online communities. The participation is anonymous and voluntary. There are no defined exclusion or inclusion criteria other than participation in the online survey and completion of the entire questionnaire. The ePatient Survey 2018 was conducted between March 1 and May 1, 2018. A total of 37,589 participants were included, of which 9664 datasets were complete and a final 9621 were evaluated. The LimeSurvey software (Hamburg, Germany) was employed to conduct the survey. The evaluation of this survey assessed the responses to the following eight questionnaire items, with multiple answers allowed, where applicable:

1. Online programs and apps for health topics: How well were you able to handle them at the beginning?
2. What was the longest time you have ever used the same app or specific online disease, treatment, or health program?
3. Precisely how did your medication app help you?
4. How exactly did your online coaching program help you?
5. You said you have used an online coaching program for your illness or an app for your medications: Do you remember where you found out about this application?
6. Imagine that someone recommends an online program or app customized for your illness/treatment. From whom would you most like to receive it?
7. Imagine that you as a patient use an online program or an app that stores all your illness data for you at all times. With whom would you want to share this data (as a patient)?
8. Imagine that an app or an online program is tailored to you and your illness, including diagnostic and treatment data. From whom would you use such an application?

These questions were categorized according to TAM into questions assessing the “perceived use” and the “perceived ease of use.”

### Data Collection and Methodological Steps

#### Digital Health Care Apps

The first step was identification of the most common types of digital health care apps, which cover all information and communication technologies in the health sector including electronic health, mobile health, telemedicine, big data, and health apps [14]. The apps can be classified into seven types: health literacy promotion, analysis and knowledge, indirect intervention, direct intervention, case history documentation, organization and management, and purchasing and preventive [15]. The content of each category is listed in Table 1.

**Table 1:** Types of digital health care. Source based on Thranberend at al [15].

Type of application	Content of application
Type 1: Promoting health literacy	Information related to health or illness concerns (eg, health portals, provider comparison portals)
Type 2: Analysis and knowledge	Point-by-point collection and evaluation of health-related information (eg, symptom checker, hearing test)
Type 3: Indirect intervention - promotion of self-efficacy, adherence, and safety	Continuous collection and evaluation of health-related information (eg, digital diaries for the chronically ill, medication-taking reminder, patient communities)
Type 4: Direct intervention - change of skills, behaviors, and conditions	Prevention or treatment (eg, online courses, tutorials, smartphones as hearing aids)
Type 5: Documentation of health and medical history	Storage and administration of data and reports (eg, electronic patient records)
Type 6: Organization and administration	Process management in the health care sector (eg, online offices, appointment scheduling)
Type 7: Purchasing and medical care	Purchasing products (eg, online pharmacies)

#### Sociodemographic Data

In the second step, sociodemographic data of the ePatient Survey 2018 was evaluated to allow for ranking of the evaluation of the respondents based on their age and educational level.

#### User Typology

The last step in the study was an assessment of the user typology and differentiation into healthy users, patients with acute conditions, and patients with chronic conditions and characterization of users versus nonusers.

#### Ethics

Ethical standards associated with social science research were applied to this research. This study aimed to interpret the data such that it reflected its original emphasis rather than the researchers’ own preferences. Moreover, the study protected the privacy of the sources of any views expressed within the study interviews by conducting the survey in an entirely anonymous fashion. At no point were any personal data pertaining to the respondent’s name or medical records obtained.

## Results

### Sociodemographic Data

The average age of the respondents in the study was 59.7 years (SD 16 years). The majority of the respondents aged <40 years were female (n=6735, 70%). In contrast, 60% (n=5773) of the respondents aged >70 years were male [13]. In terms of the educational level, 41% (n=3945) of the respondents had a university or technical college degree, 40% (n=3848) had a high-school diploma (“Realschulabschluss”) or a university-entrance diploma (“Abitur”) without higher studies, and 18% (n=1732) had a certificate of secondary education (“Hauptschulabschluss”). In an all-German comparison, the academic rate in the survey was well above average, which did not significantly affect the study’s validity [13]. In addition, 72% (n=6927) of the respondents had government insurance, while 11% (n=1058) had a private insurance and 17% (n=1636) had both.

### Frequency of Used Health Care Apps

The most common type of health care app was an online medical appointment scheduling app (n=2309, 24%), followed by tracking apps (n=1827, 19%) that record all types of data collections. Coaching apps (n=1347, 14%) and an online medical second opinion app (n=770, 8%) were also in relatively widespread use. Apps for diagnosis (n=577, 6%), check-ups (n=385, 4%), online health records (n=241, 2.5%), and online medical consultations (n=96, 1%) were less widely used [13].

### User Typology

Long-term patients with chronic diseases accounted for the greatest proportion of users (n=4041, 42%); this group consisted of predominantly male patients with an average age of 63.3 years and no academic background. The majority of those with chronic diseases (n=2966, 73.4%) were receiving treatment at the time of the survey, with 51% (n=1513) of these patients taking medication, 19% (n=564) taking part in physical therapy, and 17% (n=504) receiving regular outpatient and inpatient treatment in a clinic.

Diseases for which digital apps were employed included primarily diseases of the locomotor system (n=1948, 27%), cardiovascular diseases (n=1876, 26%), and metabolic diseases (n=1448, 20%), followed by pain syndromes (n=938, 13%), psychiatric disorders (n=866, 12%), and ophtalmological diseases (n=721, 10%). The second largest group comprised patients with acute conditions (n=3175, 33%), with an average age of 56.6 years, and this group had a higher proportion of women (n=2000, 63%). Users in this category tended to be college graduates. Healthy users made up the smallest group (n=2405, 25%), with an average age of 58.6 years and no sex-relevant tendencies; this group had a significantly higher proportion of university/college graduates [13]. Most patients were receiving ongoing treatment during the survey and thus used the internet more frequently. The second most common phase was change of treatment or desire for a change; this phase was associated with slightly increased internet usage [13].

The intensity of digital medical app use was classified into nonusers, users, and intensive users. Nonusers were defined as never having used any digital medical app due to the lack of need for or interest in such apps. People in this category showed a limited willingness to share their data and a certain mistrust toward anybody but their physician. The average age in this group was 63 years, 4 years above the overall age average of 59 years. The level of education of people in this group was slightly lower than that in the user group [13]. The user group comprised people who regularly used digital apps for medical devices, medication, coaching, and obtaining a second opinion online. People in this group had a higher level of knowledge, a higher need for an online health record, and were generally more willing to share their personal data.

Of the respondents aged ≤40 years, the majority were women, while a higher proportion of men were observed in the age group of those aged >60 years. The average age in this group of 52 years was 7 years below the average age of 59 years. This user type had a slightly higher level of education than nonusers. Intensive users exhibited the greatest need for online medical records and consequently had the highest knowledge. The most frequently used apps in this group were, similar to the user group, those for medical devices, medications, coaching, and obtaining an online second opinion. Every second person in this group had completed an academic degree [13]. The average age was the same as that in the user group (52 years)—7 years below the overall age average. A higher age correlated with a higher proportion of men.

The evaluation results of the responses to the questionnaire items are summarized in Table 2.

**Table 2:** Summary of responses to the questions of the ePatient Survey 2018.

	Questions used in the study from the ePatient Survey 2018	Evaluation of the responses
**Questions assessing the perceived usefulness according to the TAM^a^**		
	“Imagine that you as a patient use an online program or an app that stores all your illness data for you at all times. With whom would you want to share this data (as a patient)?” *(value, social support, emotion, confidence)*	In this scenario, 81% (n=7793) would like to share their data with their attending physician, 35% (n=3367) with their clinic, 28% (n=2693) with their health insurance provider, 13% (n=1251) with none of the above, and 5% (n=481) with the company producing their medication.
	“What was the longest time you have ever used the same app or specific online disease, treatment, or health program?” *(experience, usability*)	37% (n=1607) of users stopped using it after only a few days, 20% (n=869) after a few weeks, 19% (n=825) after a few months, and 24% (n=1043) used it for ≥1 year.
	“Precisely how did your medication app help you?” *(value, usability, accessibility, emotion, independence, experience, confidence)*	51% (n=306) of users said it helped them take their medication regularly, 27% (n=162) said it made no difference, 22% (n=132) saw somewhat of an improvement, 57% (n=342) saw an improvement in handling their medication, 29% (n=174) saw somewhat of an improvement, and 14% (n=84) did not see an improvement.
	“How exactly did your online coaching program help you? – I cope much better with my illness and my everyday life with the illness.” *(value, usability, accessibility, technical support, social support, emotion, independence, experience, confidence)*	33% (n=41) of online coaching program users said they were coping better with their illness in everyday life, 50% (n=63) saw somewhat of an improvement, and 17% (n=21) saw no improvement.
**Questions assessing the perceived ease of use according to the TAM**		
	Online programs and apps for health topics: “How well were you able to handle it at the beginning?” *(usability, accessibility, independence, experience, confidence)*	More than 50% (total n=4446) of users initially had minor to major difficulties operating health programs and apps, 46% (n=2045) stated that it was easy from the start, 39% (n=1734) stated that it required some experimentation and patience, and 15% (n=667) had major issues.
	“You said you have used an online coaching program for your illness or an app for your medications: Do you remember where you found out about this application?” *(accessibility, independence)*	68% (n=717) discovered the app by searching the internet themselves, 16% (n=169) received a recommendation for the app from their health insurance fund, 9% (n=95) from their physician, 8% (n=84) from family and friends, 8% (n=84) from magazines or the radio, and 5% (n=53) from their pharmacy.
	“Imagine that someone recommends an online program or app customized for your illness/treatment. From whom would you most like to get it?” *(technical support, social support, emotion, experience)*	72% (n=6927) would prefer to get this app from the attending physician, 40% (n=3848) from their health insurance provider, 20% (n=1924) would search for it by themselves on the internet, 15% (n=1443) would obtain it through a pharmacy, 13% (n=1251) from their hospital, and 8% (n=770) and 5% (n=481) from the company producing the medical device or medication, respectively.
	“Imagine that an app or an online program is tailored to you and your illness, including diagnostic and treatment data. From whom would you use such an application?” *(affordability, accessibility, technical support, social support, emotion, independence, experience, confidence)*	The majority of respondents said that they would use such an app if it came from their health insurance provider (55%, n=5292) or their physician's software (55%, n=5292). In addition, 23% (n = 2213) would use a governmental app, 12% (n=1155) one from an information technology provider in Germany and 6% (n=577), 5% (n=481), and 1.5% (n=144) from Google, Apple, and Amazon, respectively.

^a^TAM: Technology Acceptance Model.

## Discussion

### Principal Findings

The decision to integrate new technologies in the health care industry depends on different factors common to patients, medical professionals, and health care providers. Knowledge on the attitude of potential users toward such technologies eventually determines their success on the market. In this study, we therefore assessed the frequency of use of different digital medical apps in Germany to characterize the current user population and identify the perceived usefulness and ease of use of such apps.

Our most important findings and a brief discussion of each finding are presented below.

The perceived usefulness of the apps was positively supported by the statement from most patients that such an app helped them regularly take their medication. Nonetheless, most users did not use the app for an extended period of time, with less than a quarter of the respondents continuing its use for a year or more. The acceptance is therefore apparently very short-lived and may limit the potential benefits (eg, long-term apps such as storage of the medical history). The most prominent perceived usefulness is the sharing of data stored in an app with their attending physician. In contrast, online coaching programs were not perceived as a helpful tool by most participants, with only one-third noting an improvement in their daily life after participating in such a program. If the users fail to see any long-term value in online coaching programs, the probability of acceptance likely decreases. Of note, the use of such programs has increased from 5% (n=200) in 2016 to 14% (n=1485) in 2018, likely due to increasing advertisement and a broader availability of such different programs.

In terms of the perceived ease of use, we discovered that users of medical digital apps are still facing minor or major difficulties in operating them. Furthermore, there appears to be a discordance between the preferred access to such apps (ie, the attending physician) and the actual way such apps are accessed (ie, the internet). At the same time, nonusers frequently showed a certain distrust toward people administering such apps other than their physicians. In addition, 72% (n=6927) would prefer to receive this app from the attending physician. This shows a clear divergence between the distribution channels actually used and the distribution channels desired.

Thus, the physician is clearly preferred to other parties (such as a hospital, health insurance fund, and medication manufacturer). User confidence is a crucial factor in the long-term acceptance of new technologies. There is generally a relationship of trust between the patient and the (family) physician that is sometimes built up over several years. To make the introduction of innovative technologies sustainable and promising for the user, it is advisable, based on current data, to have the attending physician function as the direct distribution channel.

An online health record system could facilitate the documentation of a patient’s case history and prove to be cost-effective. Such a record system, the Siemens patient records system, was used in Austria as part of the digiLog project [16]. In this system, patient data were stored as a mobile electronic file and could be shared between the attending physicians and the patient [16]. However, the online health record has been receiving a low participation to date (n=241, 2.5%), possibly due to its pilot project status due to limited health insurance funds, making it accessible only to privately insured patients [13]. Its nationwide use requires the right framework, such as a basic social understanding of the benefits of such a record system and strategies for building and disseminating it [12].

In our study population, there was a low level of awareness of online patient record systems among female respondents and an apparent lack of interest in younger male respondents with a higher level of education. We speculate that a low level of awareness might prevent patients from using the available apps. An app’s acceptance is generally linked to awareness of its capabilities. Hence, an increased promotion of digital medical apps, ideally by the person of trust, namely, the physician, could potentially increase their acceptance among patients.

Acceptance must be further differentiated according to sociodemographic factors and the types of the respective apps [17,18]. A recent study has shown relatively lower acceptance rates in older individuals [19]. Respondents in this study were 59 years old on average, with most of the respondents aged between 44 and 76 years. An extrapolation of our results to a younger population is therefore not possible, and we cannot judge the acceptance in younger adults in our cohort.

Several recent reports have addressed the acceptance of digital health technologies in specific cultural and socioeconomic settings. A pilot study in female respondents in rural Uganda has shown overall openness among the respondents to accept computer-assisted personal interviewing. The implementation of such a technology can, in the long run, be very beneficial for the community because it would provide a cost-effective and more easily accessible health care alternative [20]. It appears crucial that the anxieties and insecurities of both patients and medical professionals are considered to increase the chances for their acceptance [21].

Digital medicine has great potential to bring individualized health care solutions, but challenges for their successful implementation still exist. Many apps still need to be validated in clinical settings and, often, initial pilot studies may be underpowered [22]. Ethical challenges also need to be addressed, such as concerns about the confidentiality of personal data [23,24].

The survey revealed significant differences in the current spread, use, and perception of digital health technologies among users in Germany. Suitable framework conditions must be generated, particularly in the case of groups that currently still show a low level of acceptance of specific apps (including online patient records and online second opinions). Such conditions must take into account socioeconomic aspects and the use of apps by the younger generation as well as sex differences. Patients must also be informed about the capabilities of the app and a suitable offer must be made available.

The obvious limitation of our study is the average age of the respondents—59 years. This can most likely not be considered representative of the German population as a whole, which had a median age of 47.1 years in 2018 [25]. The reason for this high average age can be attributed to the type of channels through which the ePatient Survey was distributed, namely, health-relevant websites and newsletters, whose readership is typically older.

### Conclusions

Our evaluation demonstrates the acceptance of medical digital technologies among a selected population group in Germany, primarily as tools to communicate with the attending physician. Our findings highlight the need to generate a framework for such technologies by increasing the knowledge on their existence and benefits and supporting them with respective funds from the health care providers.

## Abbreviations

CAPI: computer-assisted personal interviewing
EMR: electronic medical record
TAM: Technology Acceptance Model
UTAUT: Unified theory of acceptance and use of technology
